# Overlapping states of AWGS muscle dysfunction and inverse feasibility of ADL recovery by rehabilitation in older inpatients

**DOI:** 10.1038/s41598-022-26622-z

**Published:** 2022-12-24

**Authors:** Masashi Yamashita, Hiroaki Obata, Kentaro Kamiya, Atsuhiko Matsunaga, Kazuki Hotta, Tohru Izumi

**Affiliations:** 1 Department of Rehabilitation Sciences, Kitasato University Graduate School of Medical Sciences, 1-15-1 Kitasato, Minami-Ku, Sagamihara, Kanagawa 252-0373 Japan; 2 Division of Research, ARCE Inc, Sagamihara, Japan; 3 Division of Rehabilitation, Niigata Minami Hospital, Niigata, Japan; 4 Department of Rehabilitation, Kitasato University School of Allied Health Sciences, Sagamihara, Japan; 5 Kitasato University, Tokyo, Japan

**Keywords:** Geriatrics, Quality of life, Rehabilitation

## Abstract

Rehabilitation based on guided walking is effective to recover activity of daily living (ADL) in frail older adults, even octogenarians. However, muscle dysfunction obviously reflects disability, and few studies have focused on ADL recovery by rehabilitation. We employed the state of muscle dysfunctions proposed by the Asian Working Group for Sarcopenia (AWGS) in 2019 and attempted to clarify the relation between the overlapping dysfunctions and the feasibility of ADL recovery after rehabilitation. In total, 297 frail older patients (the mean age: 82.8 years, 46.1% of patients were male) participated in a walking-guided rehabilitation program to achieve the goal of ambulatory discharge. Muscle dysfunction was categorized by four standardized methods at the start of rehabilitation (grip strength, gait speed, time of five sit-to-stand, and short physical performance battery: SPPB), according to the AWGS proposal. ADLs were monitored by Barthel index before admission, at the start of rehabilitation, and at discharge. At least one dysfunction was present in 95.3% of patients. If a single patient had three or more muscle dysfunction, the ADLs recovery was significantly limited (interaction: *p* < 0.05). The overlapped counts of AWGS muscle dysfunction helps to predict inverse feasibility of ADL recovery in frail older patients through rehabilitation.

## Introduction

Around the world, but especially in Japan, the number of super-aged individuals is increasing^1^. Awareness of this serious issue among those in the medical profession has increased each year^2^. In Japan, the majority of patients admitted to community-based hospitals are octogenarians, and most exhibit reduced activities of daily living (ADL), regardless of the underlying causes of the disease. Lower ADL is directly related to prognosis^3^. In addition, hospitalization itself is the main factor in reducing ADL in the frail older adults^4^. Therefore, rapid inpatient rehabilitation for older patients is essential to improve their ADL and to clarify the feasibility of ADL recovery to return to their way of life before admission. However, the disease conditions and physical activity of the older adults are widely diverse, especially in octogenarians, and rehabilitation requires special preventive care of falls and/or bone fracture as risk management and cardiovascular events as disease management. Thus, since 2013, we have been developing a hospital rehabilitation based on a walking guide to achieve the goal of ambulatory discharge in the older patients, which we have termed DOPPO rehabilitation: Discharge Of older Patients from hosPital On the basis of their independent gait program^5^. To date, this project has been used to meet the needs of a super-aged society and has reached a paradigm in which most of the rehabilitative frail older can sufficiently recover their ability for ambulatory discharge^3,5^.

Undoubtedly, a state of muscle dysfunction reflects disability, but few studies have focused on the feasibility of ADL recovery by rehabilitation so far. To determine the cases of muscle dysfunction that could feasibly recover the ADLs by rehabilitation remains a cardinal problem to be solved. Presently there are many available muscle function tests, but none provide definitive diagnosis. From a practical point of view, the ideal test should be easy and straightforward and open to anyone, anywhere, and anytime. Further, its credibility should be highly evidenced. For example, it is well known that gait speed is a marker for all human prognoses^6^, and grip strength is a representative measure of frailty^7,8^. Thus, gait speed is one of the critical indices to judge the effectiveness of rehabilitation, and some investigators have emphasized that rehabilitation guided by gait speed can improve not only muscle function, but also the ADLs and their prognosis^5,9,10^.

In 2019, a new set of criteria for diagnosing possible sarcopenia were proposed by the Asia Working Group for Sarcopenia (AWGS)^11^. The AWGS introduced a new concept of muscle dysfunction as possible sarcopenia, defined by only patient status (calf circumference) and four functional muscle tests (grip strength, gait speed, the time interval of five sit-to-stand exercises, and short physical performance battery: SPPB). These tests do not require the use of any advanced or special equipment, making diagnosis easy in practice. The AWGS intends to widely use the diagnosis of possible sarcopenia to promote health care for super-aged individuals and minimize the social burden associated with long-term nursing care. The proposed tests are already familiar to any personnel working in rehabilitation, as they are routinely used. However, the utility of this a diagnostic tool for rehabilitation has not been elucidated, and evidence of its scalability is insufficient.

Thus, we employed the four AWGS tests for possible sarcopenia to establish a diagnosis of the muscle dysfunction states and elucidated the relationship between the overlapping states of muscle dysfunction and the feasibility of ADLs recovery after rehabilitation.

## Results

### Patients flow and characteristics

**Figure 1:**
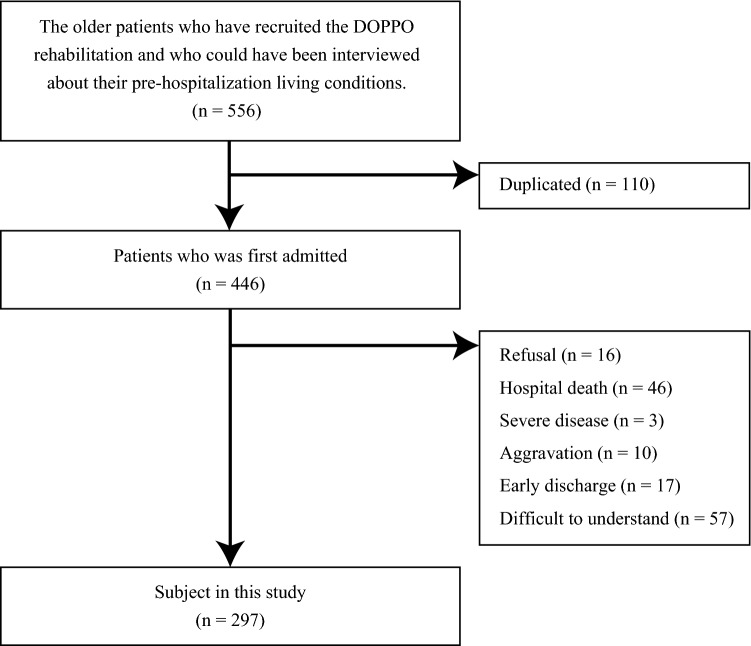
The patients flow in this study.

Finally, data of 297 consecutive patients were extracted out of 556 sequentially admitted candidates who participated DOPPO project (Fig. 1). The baseline characteristics of all subjects are shown in Table 1. The mean age of the older patients enrolled in this study was 82.8 years, and 71.4% (212/297) of the patients were octogenarian. The Barthel index (BI) scores before admission, at the start of rehabilitation, and at discharge were 77.7, 54.4, and 73.6, respectively. Further, the mean changes in the BI score between pre-hospitalization and the start of rehabilitation and between before and after rehabilitation were − 23.3 and 19.2, respectively. Usual gait speed, grip strength, five sit-to-stand exercises, and SPPB were 0.73 m/s, 18.8 kg, 17.56 s, and 7.3 points, respectively. At least one muscle dysfunction (low grip strength, slow gait speed, slow time interval of five sit-to-stand exercises, or low score of SPPB) was present in 95.3% of all individuals, even after imputation.

**Table 1:** Patient characteristics in this study. Data are presented as mean ± standard deviation unless otherwise indicated. BMI, body mass index; eGFR, estimated glomerular filtration rate; SPPB, short physical performance battery.

Characteristics	Overall n = 297		
Age, year	82.8	±	7.6
Age over than 80 years (%)	212		(71.4)
**Sex**			
Male (%)	137		(46.1)
Female (%)	160		(53.9)
BMI, kg/m^2^	21.0	±	2.8
Hypertension (%)	196		(66.0)
Diabetes mellitus (%)	107		(36.0)
Dyslipidemia (%)	80		(26.9)
**Barthel index, points**			
Before admission	77.7	±	28.4
Before inpatient rehabilitation	54.4	±	36.1
After inpatient rehabilitation	73.6	±	28.2
**Change in Barthel index, points**			
Between before admission and before inpatient rehabilitation	−23.3	±	45.2
Between before and after the inpatient rehabilitation	19.2	±	28.4
Hemoglobin, mg/dL	11.6	±	2.4
Albumin, mg/dL	3.57	±	0.50
eGFR, mL/min/1.73 m^2^	55.4	±	23.2
White blood cell, mg/dL	7710.8	±	4600.4
Mini-mental state examination, points	21.9	±	4.2
C-reactive protein, mg/dL	1.05	±	0.63
Gait speed, m/s	0.73	±	0.23
Grip strength, kg	18.8	±	6.0
Time interval of 5 sit-to-stand exercises, sec	17.56	±	8.02
SPPB, points	7.3	±	3.1
One or more dysfunctions (%)	283		(95.3)

### Results of overlapping muscle dysfunctions

Table 2 shows the overlapping dysfunctions and prevalence according to underlying causative disease for all participants and octogenarians. For every type of disease, more than 90% of the older patients had one or more overlapping dysfunctions, but this was not statistically significant (all older patients, *p* = 0.308), nor was the sensitivity analysis (octogenarian: *p* = 0.154).

**Table 2:** Prevalence of dysfunction and overlapping of dysfunctions according to the primary disease. *Statistically significant (p < 0.05). CVD, cardiovascular disease.

	CVD	Lung	Orthopedic	Cancer	Cerebral	Kidney	Other	*p* value*
[All cases]	n = 89	n = 49	n = 38	n = 43	n = 11	n = 12	n = 55	
One or more dysfunction	85 (95.5)	46 (93.9)	37 (97.4)	40 (93.0)	9 (81.8)	12 (100.0)	54 (98.2)	0.308
[Age over than 80 years old]	n = 68	n = 38	n = 28	n = 25	n = 5	n = 10	n = 38	
One or more dysfunction	67 (98.5)	37 (97.4)	28 (100.0)	23 (92.0)	4 (80.0)	10 (100.0)	37 (97.4)	0.154

**Figure 2:**
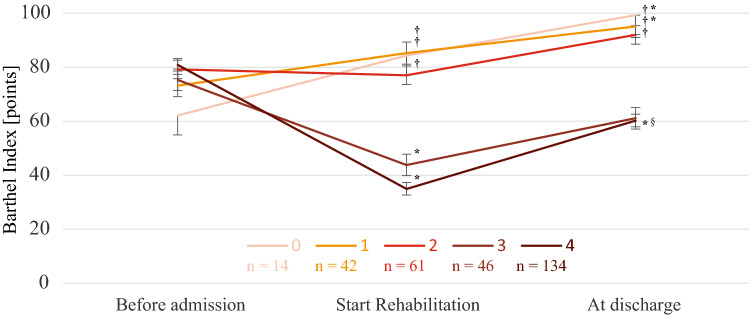
Association between overlapping muscle dysfunctions and the ability to perform activities of daily living. The number of subjects in each group is indicated by “n”. * There was a significant difference in the ADLs before and after admission (*p* < 0.05). †There was a significant difference in the ADLs between 3 or more and less than 3 with overlapping muscle dysfunctions (*p* < 0.05). § There was a significant difference in the ADLs before and after inpatient rehabilitation (*p* < 0.05). ADLs, activities of daily living.

The results of the two-way analysis of covariance (two-way ANOVA) are shown in Fig. 2. There was a significant interaction between the BI trajectory and overlapping dysfunctions (F-value: 17.36, *p* < 0.001). Post hoc analyses were performed as both the trajectory of the BI (before admission, before and after rehabilitation, F-value: 16.69, *p* < 0.001) and the overlapping dysfunctions (from 0 to 4, F-value: 43.31, *p* < 0.001) had significant effects. The BI value before rehabilitation decreased significantly compared to that before admission for the patients who had three or four overlapping dysfunctions (*p < 0.05), and only the group of four overlapping dysfunctions. Conversely, the group of 0 or 1 overlapping dysfunctions showed improved ADLs compared to before admission. Moreover, compared to patients with less than two overlapping dysfunctions, the older patients with three or four overlapping dysfunctions had a lower BI before and after rehabilitation (†*p* < 0.05). However, rehabilitation significantly improved ADLs in the group with four overlapping muscle dysfunctions (§*p* < 0.05). These trends were similar in all sub-analyses (Supplementary Fig. 1). Furthermore, all older patients in the group with one or two overlapping muscle dysfunctions were discharged from the hospital walking independently on their own feet (BI gait item ≥ 10 points).

### Factors associated with overlapping muscle dysfunctions

The results of the Poisson regression analysis are shown in Supplementary Table 1 with incident rate ratio (IRR) and 95% confidence interval (CI) limit. The BI before admission was significantly associated with overlapping dysfunctions until model 3 (IRR: 0.002, 95% CI limit: 0.000 to 0.004, *p* = 0.038), and tended to be significant, even in model 4 (IRR: 0.002, 95% CI limit: 0.000 to 0.004, *p* = 0.086). Like other factors, age (IRR: 0.020, 95% CI limit: 0.012 to 0.027, *p* < 0.001) and albumin (IRR: − 0.154, 95% CI limit: − 0.270 to − 0.037, *p* = 0.010) were significantly associated with overlapping dysfunctions. Hemoglobin showed a tendency towards statistical significance (IRR: 0.024, 95% CI limit: − 0.001 to 0.049, *p* = 0.061).

### Components of muscle dysfunction associated with ADL recovery through rehabilitation

The results of the multiple regression analysis after adjusted for confounding factors are shown in Table 3. At the single component, lower grip strength (estimate value: 12.60, 95% CI 4.79–20.40, t-statistics: 3.18) or slower sit-to-stand time (estimate value: 8.98, 95% CI 1.76–16.20, t-statistics: 2.45) at the start of rehabilitation was associated with better recovery of BI scores. In the combination of the two components, the combination of grip strength and sit-to-stand was significantly related to ADL recoverability (estimate value: 11.69, 95% CI 3.30–20.08, t-statistics: 2.74, β: 0.296). The *t* statistic was largest for all constructs (estimate value: 7.71, 95% CI 3.75–11.68, t-statistics: 3.83).

**Table 3:** The relationship between change in Barthel index score from before to after inpatient rehabilitation and components of muscle dysfunction (low grip strength, slow gait speed, and slow time of sit-to-stand) using multiple regression analysis. Adjusted by Barthel index score before admission, age, sex, body mass index, Mini-Mental State Examination, hypertension, dyslipidemia, diabetes mellitus, albumin, hemoglobin, white blood cell, estimated glomerular filtration rate, and C-reactive protein. CI, confidence interval; ES, estimate value.

Adjusted by confounding factors	ES	95% CI			t value	β	*p* value
**[Single component]**							
Low grip strength (vs. retain)	12.60	4.79	–	20.40	3.18	0.197	0.002
Slow gait speed (vs. retain)	−1.81	−12.76	–	9.14	−0.32	−0.020	0.746
Slow time of sit-to-stand (vs. retain)	8.98	1.76	–	16.20	2.45	0.155	0.015
**[Two components]**							
Low grip strength + slow gait speed	0.91	−7.21	–	9.02	0.22	0.018	0.826
Low grip strength + slow time of sit-to-stand	11.69	3.30	–	20.08	2.74	0.296	0.007
Slow gait speed + slow time of sit-to-stand	−2.71	−10.25	–	4.83	−0.71	−0.064	0.479
**[All Components]**							
Low grip strength + slow gait speed + slow time of sit-to-stand	7.71	3.75	–	11.68	3.83	0.237	< 0.001

## Discussion

This study focused on the utility of the muscle dysfunction definition proposed by the AWGS aimed to examine the feasibility of ADL recovery by rehabilitation. First, among the frail older patients who participated in DOPPO rehabilitation to achieve the goal of ambulatory discharge, 95.3% of the patients had one or more muscle dysfunctions. Second, the degree of muscle dysfunction was comparable regardless of the underlying diseases. Third, older individuals with two or less muscle dysfunctions regained their previous ADL level after rehabilitation. Conversely, older individuals with three or more overlapping muscle dysfunctions showed slight and limited improvements in ADL execution ability after rehabilitation, which was not sufficient to reach the previous ADLs level. Fourth, the Poisson regression analysis indicated that the ability to perform ADLs before admission might be inversely related to the count-number of overlapping muscle dysfunctions. Moreover, age and albumin concentration were extracted as factors associated with overlapping muscle dysfunction, and hemoglobin also tended to be associated. Finally, multiple measures of muscle dysfunction were shown to be important in predicting the inverse feasibility of ADL recovery. These findings indicate that a total assessment of muscle dysfunction involving multiple aspects is required for frail and older patients, regardless of the disease causes.

This study found that whether patients might return to their pre-hospital ADL life could be inversely determined by the overlapping states of the AWGS muscle dysfunction, and that ADLs might be sufficiently improved if the number of muscle dysfunction is within two. Moreover, a combination of all components of muscle dysfunction was associated with improvement in BI scores. The REHAB-HF^12^, one of the most crucial intervention studies in frail and older patients in recent years, reported a sufficient effect (approximately three times the minimum clinically significant difference in SPPB) in patients with heart failure with 4–9 points of SPPB requiring adequate risk management at the time of intervention. Furthermore, the incremental cost-effectiveness ratios determined by quality-adjusted life year in the REHAB-HF study confirmed more beneficial findings in patients with preserved left ventricular ejection fraction, who are generally more likely to occur in older women^13^. Similarly, in this study, even the most severe muscle dysfunction patients benefited from rehabilitation and improved about 20 points in BI. These findings suggest that even octogenarians with severe muscle dysfunction may benefit from hospital rehabilitation to achieve the goal of ambulatory discharge.

We quantitatively evaluated muscle function in the present study using the AWGS proposal^11^. Muscle function assessment often requires the competence of professionals in terms of time measurement and setting of measurement conditions. Therefore, seamless and consistent evaluation from the hospitalization is desirable, and in this study, we adopted the performance tests of AWGS criteria, which are easy and straightforward and can be used anywhere and anytime. Conversely, the evaluation of muscle mass was omitted here. Indeed, although muscle mass assessments are important to understand the patient's body condition, their measurement is still controversial in the definition of sarcopenia in no small way^14,15^. Currently, a new Global Leadership Initiative on Sarcopenia is being launched to reach an international consensus on the definition of sarcopenia, with discussions focusing on muscle function such as muscle strength rather than muscle mass^16^. In practice, the performance assessment is prioritized over the muscle mass assessment, as is also the case in our hospital. Muscle function is modifiable, although it is difficult to improve muscle mass in the older population^17^. Within the limited time frame of hospitalization, interventions aimed at improving muscle performance are more urgent and directly related to ADL recovery for the older patients than interventions aimed at increasing muscle mass. In fact, it was suggested that the higher the number of muscle dysfunctions, the more likely it was that the BI score would improve with subsequent rehabilitation in the present study. In addition, it has been mentioned that even measurement using medical imaging, which is considered the gold standard for muscle mass assessment, is prone to errors due to edema or other effects^18^. Moreover, some special machines require the older patients to enforce the measurement posture firmly, which is often difficult to measure in individuals with severe frailty or limited ADL. Therefore, we have been adopting the approach that quantitative evaluation for muscle mass should be the next step after ADL and muscle function improvement.

Age was strongly associated with the number of overlapping muscle dysfunctions themselves, and albumin and hemoglobin were also associated. Albumin^19^ and hemoglobin^19,20^ are considered markers of malnutrition. Therefore, the malnutrition status of the older adults might have been closely related to dysfunction. Exercise interventions and other professionals, including nutritionists, may be requested to effectively rehabilitate the older patients with overlapping muscle dysfunctions.

The BI improved by about 20 points in the present study in all rehabilitative older patients, confirming the usefulness of hospital rehabilitation even in the group with the most overlapping muscle dysfunction. Conversely, older patients with a high degree of overlapping states of muscle dysfunction showed more significant improvement in BI score through inpatient rehabilitation, but insufficient improvements in the ADL score compared to before admission. We previously reported similar data obtained from older patients with heart failure^3^. These results suggest that it is essential for every older individual to set a reasonable goal regarding their ADLs, using feasible rehabilitation, before admission. Reasonable goal setting and following the easy-to-understand outcomes it will support comprehensive rehabilitation activities, and such efforts will realize a reasonable burden acceptable in a super-aged society.

This observational study has several limitations. First, the causal relationship between overlapping states of AWGS muscle dysfunction and the inverse feasibility of ADL recovery by rehabilitation for the older patients cannot be clearly asserted because of the retrospective nature of this study. Next, a certain number of older adults did not undergo initial measurement of muscle functions, despite every effort being taken. This is a somewhat unavoidable event in studies on older adults according to their mental and physical conditions. While the same is applied to the evaluation of pre-symptomatic BI scores, the reliability and validity of the BI score based on pre-symptomatic ADL interviews remains a concern. However, the present study represents a valuable report composed of a population with a high percentage of octogenarians. Since there was little difference in the results before and after additional statistical imputations, these results have practical validity. Furthermore, the fact that there was a clear difference in the improving process of ADLs according to the degree of muscle dysfunctions is one of the strengths of the present study, even considering these limitations. The inverse feasibility of ADL recovery related to the overlapping states of muscle dysfunction should be more emphasized from now on. Lastly, the present study was limited to in-hospital observations. Therefore, further examinations of patient progress after hospital discharge are required, and it will be necessary to verify such concordance using a prospective study.

In conclusion, although muscle dysfunction was ordinally found in most frail older patients, regardless of diseases causes, a clear difference in the inverse feasibility of ADL recovery by the comprehensive rehabilitation was observed depending on the states of dysfunction. Furthermore, these results were consistent even in octogenarians. The results of this study would indicate the further need for a multifaceted and comprehensive evaluation of muscle dysfunction. Moreover, the present findings help provide much refrained rehabilitative resources to facilitate the early recovery to ADLs level before admission.

## Methods

### Study population and hospital rehabilitation for older adults

We retrospectively reviewed a cohort of all patients ≥ 65 years who were sequentially admitted to the Niigata Minami Hospital due to any disease and discharged after the end of rehabilitation in the DOPPO project between anuary 2017 and April 2019, for whom data on ADLs were collected before admission, at the start of rehabilitation, and at discharge. Patients who refused to cooperate with measurements of muscle functions for any reason were excluded. During rehabilitation, their physical frailty was measured by the SPPB score. The present study was a cross-sectional observational study of DOPPO rehabilitation approved by the Research Ethics Committee of Niigata Minami Hospital (no. 1502) and was conducted in accordance with the Declaration of Helsinki. Because the study protocol was based on anonymous extraction and review of medical records, the institutional review board waived the need for patient consent. An information about the research was made public by opt-out, and the participants were informed of the option to drop out.

Here, we will briefly introduce the content of the DOPPO project; more detailed descriptions have been provided previously^5^. This project aims to rehabilitate frail and older patients (most of them octogenarian, average age 82 years) who are objectively at risk of losing their independent walking ability, such as inability to go to the toilet and to take a meal on admission, but whose physical frailty is expected to be plastic and reversible from their ADL state before admission. Goal setting is straightforward, with rehabilitation aimed at achieving ambulatory discharge, to enjoy an independent life and to recover the ADL level similar to pre-admission. The rehabilitative program is comprehensive, but with only four components: stretch gymnastics, muscle strengthening, balance training, and endurance. Each program is guided by walking ability in a step-by-step manner. Practically, important points are monitored with SPPB, 10 m walking speed, and gait style. The risk is managed according to the guideline of cardiac rehabilitation^21^ (Supplementary Fig. 2). Under this comprehensive rehabilitation, approximately one month of hospital stay improves the ADLs of older adults; overall, the average 10 m walking speed increases from 0.8 to 1.0 m/sec, and the SPPB score is improved from 7 to 9 points, on average^5^. Accordingly, approximately 80% of patients are discharged to their previous residence, walking independently, having met the goals. From such project outcomes, we consider that the DOPPO rehabilitation is a valuable measure for frail and older patients and a suitable method to verify the feasibility of ADL recovery.

### Clinical data collection

Data for all the evaluated variables were extracted from electronic medical records. Height was recorded to the nearest 0.1 cm, and a calibrated scale was used to determine the weight to the nearest 0.1 kg. Body mass index (BMI) was calculated as the body weight (kg) divided by height (m^2^)^22^. The causative underlying disease and comorbidities were collected from medical insurance codes and the diagnosis procedure combination (DPC) disease name. Data regarding hemoglobin, albumin, white blood cells and C-reactive protein were collected at admission. The Japanese Society of Nephrology formula was adapted to define the estimated glomerular filtration rate (eGFR)^23^.

The ADLs were assessed by a nurse or rehabilitation therapist, and identified by the BI^24,25^ at the start of rehabilitation and at discharge. Additionally, we obtained data before admission from the patients as early as possible after admission. If necessary, the family member or someone who could closely understand his/her living situation were also questioned^26,27^. The BI is scored in increments of 5 points (highest possible total score = 100). The values assigned to each item are weighted according to the amount of physical assistance required if the patient cannot perform the activity independently. The 10 ADLs assessed by the BI were as follows: feeding; wheelchair transfer to and from the bed; personal hygiene; toilet use; bathtub use; walking (wheelchair management if the patient is non-ambulatory); ascending and descending stairs; dressing; bowel control; and bladder control.

### Measurements and definitions of muscle dysfunction

We measured muscle functions, such as muscle strength (grip strength) and physical function (usual gait speed, the time interval of five sit-to-stand, and SPPB) as soon as possible after hospitalization according to the AWGS 2019 criteria^11^. Grip strength was measured using a digital dynamometer (TKK 5101 Grip-D; Takei, Tokyo). When performing dynamometry, the patients stood with their elbow joint extended. The participant gripped the dynamometer gradually and continuously for 3 s. The highest strength values on the right and left sides were averaged and expressed as an absolute value. To measure the usual gait speed, patients were asked to walk at their usual speed for 10 m of a 13-m walkway while they were timed. The SPPB, comprehensive lower limb function index, consisted of three components, usual gait speed to 4-m, repeated chair stands, and standing balance; these were measured using established methods and assigned a total score from 0 to 12, with a maximum of 4 points possible for each component (range: 0 = worst and 12 = best)^28^. Repeated chair stands were also used to determine the time interval of five sit-to-stand exercises.

The presence of muscle dysfunctions was diagnosed according to the proposal of the AWGS based on usual gait speed < 1.0 m/s, grip strength < 28.0 kg for men and < 18.0 kg for women, the time interval of five sit-to-stand exercises of 12.0 s or more, and SPPB score of ≤ 9 points^11^. Based on these indices, the degree of muscle dysfunction was defined as overlapping dysfunction from minimum 0 to maximum 4.

### Statistical analysis

Continuous variables are expressed as the means ± standard deviation, and categorical variables are expressed as number and percentage. Imputation was performed to generate the dataset of the complement missing values by using multivariate singular value decomposition imputation for JMP (Stata Corp LP, College Station, TX, USA)^29^. We used a post-imputation dataset for primary analyses. First, the chi-squared test was performed to investigate the difference in dysfunction incidence according to the primary disease. Then, a two-way ANOVA was performed with the BI as a dependent variable. The results are shown as the mean and standard error. When an interaction was recognized between the measurement time of the BI and overlapping dysfunction, and when the main effect was recognized for both the measurement time of the BI and overlapping dysfunction, Tukey’s multiple comparisons were performed as a post hoc analysis. Next, a sensitivity analysis was performed to validate the post-imputation dataset for octogenarians (age ≥ 80) and the pre-imputation dataset for all cases. Subsequently, we performed a Poisson regression analysis to examine the factors associated with overlapping dysfunction. The verified factors were as follows: model 1 = BI before admission to determine the relationship with living conditions before admission; model 2 = model 1 + age, sex, BMI, and Mini-Mental State Examination; model 3 = model 2 + hypertension, dyslipidemia, and diabetes mellitus; and model 4 = model 3 + albumin, hemoglobin, white blood cell, eGFR, and C-reactive protein. The results show the IRR and 95% CI limit. Finally, a multiple regression analysis was conducted using the change in BI from the start of rehabilitation to the time of discharge as the dependent variable to test which components of muscle function assessment (grip strength, gait speed, and StoS) were useful in determining the possibility of recovery of ADLs. Results are presented as estimates (95% CI), t-statistics, and standardized coefficient β, and the adjustment variables were the same as in model 4 of the Poisson regression analysis. Statistical analyses were performed using JMP (version 15.1; SAS Institute Inc., Cary, NC, USA) and R version 4.0.2 (R Foundation for Statistical Computing, Vienna, Austria). In all analyses, a two-tailed *p* < 0.05 indicated statistical significance.

## Supplementary Information

Supplementary Information.

## Data Availability

The datasets used this study are available from the corresponding author on reasonable request.

## Abbreviations

ADL: Activities of daily living
ANOVA: Analysis of variance
AWGS: Asia working group for sarcopenia
BMI: Body mass index
BI: Barthel index
CI: Confidence interval
DOPPO: Discharge Of older Patients from hosPital On the basis of their independent gait program
DPC: Diagnosis procedure combination
eGFR: Estimated glomerular filtration rate
IRR: Incident rate ratio
SPPB: Short physical performance battery
