# Supplementary material for: Overlapping states of AWGS muscle dysfunction and inverse feasibility of ADL recovery by rehabilitation in older inpatients
Source: Sci Rep. 2022 Dec 24;12:22283. doi: 10.1038/s41598-022-26622-z (PMC9789953; doi:10.1038/s41598-022-26622-z)
Supplement: Supplementary file 1 — Supplementary Information. [file 41598_2022_26622_MOESM1_ESM.docx]

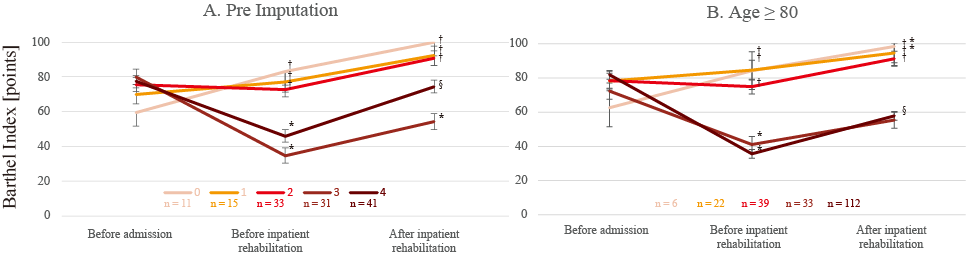


Supplementary Figure 1

The association of overlapping of muscle dysfunctions and the course of activities of daily livings according to the dataset before the statistical imputation (A) and the age 80 years or older (B).

Supplementary Table 1: Examination of factors associated with overlapping muscle dysfunctions

|  | Model 1 | | | | |  | Model 2 | | | | |  | Model 3 | | | | |  | Model 4 | | | | |
| --- | --- | --- | --- | --- | --- | --- | --- | --- | --- | --- | --- | --- | --- | --- | --- | --- | --- | --- | --- | --- | --- | --- | --- |
|  | IRR | Limited CI | | | *p* value |  | IRR | Limited CI | | | *p* value |  | IRR | Limited CI | | | *p* value |  | IRR | Limited CI | | | *p* value |
| BI before admission | 0.002 | - | - | - | 0.026* |  | 0.002 | 0.000 | - | 0.004 | 0.033* |  | 0.002 | 0.000 | - | 0.004 | 0.038* |  | 0.002 | 0.000 | - | 0.004 | 0.086 |
| Age |  |  |  |  |  |  | 0.020 | 0.013 | - | 0.027 | <.0001* |  | 0.020 | 0.013 | - | 0.027 | <.0001* |  | 0.020 | 0.012 | - | 0.027 | <.0001* |
| Female |  |  |  |  |  |  | 0.014 | -0.037 | - | 0.065 | 0.587 |  | 0.015 | -0.037 | - | 0.066 | 0.579 |  | 0.022 | -0.033 | - | 0.077 | 0.435 |
| BMI |  |  |  |  |  |  | -0.023 | -0.042 | - | -0.005 | 0.013* |  | -0.023 | - | - | - | 0.017* |  | -0.016 | -0.036 | - | 0.004 | 0.107 |
| MMSE |  |  |  |  |  |  | -0.005 | -0.016 | - | 0.007 | 0.434 |  | -0.005 | -0.017 | - | 0.007 | 0.427 |  | -0.007 | -0.019 | - | 0.005 | 0.256 |
| Hypertension |  |  |  |  |  |  |  |  |  |  |  |  | 0.012 | -0.041 | - | 0.067 | 0.651 |  | 0.010 | -0.044 | - | 0.065 | 0.711 |
| Dyslipidemia |  |  |  |  |  |  |  |  |  |  |  |  | -0.026 | -0.099 | - | 0.050 | 0.502 |  | -0.020 | -0.093 | - | 0.056 | 0.610 |
| Diabetes mellitus |  |  |  |  |  |  |  |  |  |  |  |  | 0.000 | -0.089 | - | 0.093 | 0.993 |  | -0.015 | -0.105 | - | 0.078 | 0.742 |
| Albumin |  |  |  |  |  |  |  |  |  |  |  |  |  |  |  |  |  |  | -0.154 | -0.270 | - | -0.037 | 0.010* |
| Hemoglobin |  |  |  |  |  |  |  |  |  |  |  |  |  |  |  |  |  |  | 0.024 | -0.001 | - | 0.049 | 0.061 |
| White blood cells |  |  |  |  |  |  |  |  |  |  |  |  |  |  |  |  |  |  | 0.000 | 0.000 | - | 0.000 | 0.107 |
| eGFR |  |  |  |  |  |  |  |  |  |  |  |  |  |  |  |  |  |  | 0.000 | -0.004 | - | 0.003 | 0.923 |
| CRP |  |  |  |  |  |  |  |  |  |  |  |  |  |  |  |  |  |  | -0.031 | -0.170 | - | 0.098 | 0.649 |

Model 1: Barthel index before admission; Model 2: model 1 + age, sex, body mass index, and MMSE; Model 3: model 2 + hypertension, dyslipidemia, and diabetes mellitus; Model 4: model 3 + albumin, hemoglobin, white blood cells, estimated glomerular filtration rate, and CRP.

*Statistically significant (p < 0.05).

BI, Barthel index; BMI, body mass index; CI, confidence interval; CRP, C-reactive protein; eGFR, estimated glomerular filtration rate; IRR, incident rate ratio; MMSE, Mini-Mental State Examination.


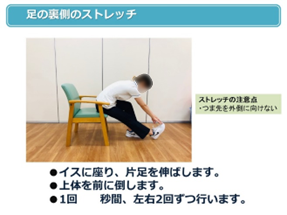

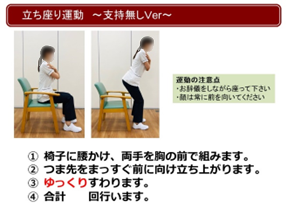

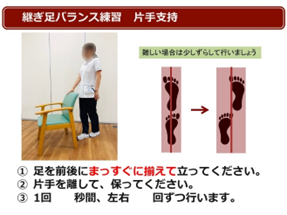


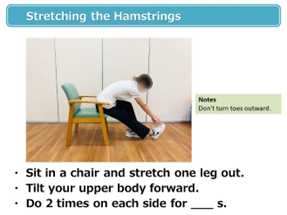

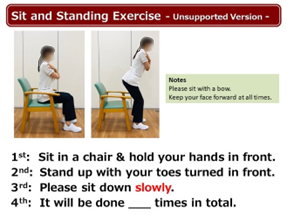

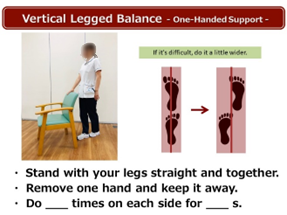


Supplemantary Figure 2

Part of semi-structured exercise cards. Based on the muscle function assessment at the time of admission and each patient's goal setting, the rehabilitation staff mainly prescribed the exercise for each patient. These images were created originally by Niigata Minami Hospital. Informed consent was obtained from all staff members appearing in the image before it was published. Also, some of the images have been processed so that individuals cannot be identified.
